# Efficacy of a Tibia Counter Rotator System for the Treatment of Internal Tibial Torsion in Children

**DOI:** 10.3390/children9070970

**Published:** 2022-06-29

**Authors:** Sungmi Kim, Mitsuyoshi Suzuki, Kei Minowa, Hiroshi Nittono, Toshiaki Shimizu

**Affiliations:** 1 Department of Pediatrics, Faculty of Medicine, Juntendo University, Tokyo 113-8421, Japan; sontarou36@gmail.com (S.K.); kminowa@juntendo.ac.jp (K.M.); tshimizu@juntendo.ac.jp (T.S.); 2 I-Leg Clinic, Tokyo 101-0062, Japan; 3 Junshin Clinic, Bile Acids Institute, Tokyo 152-0011, Japan; bile-res@eco.ocn.ne.jp

**Keywords:** in-toeing gait, transmalleolar angle, toe-out gait plate, knee joint diseases

## Abstract

Internal tibial torsion is more common in the Asian population than in Western populations. Generally, surgery should be considered for the treatment of severe internal tibial torsion. As an alternative approach, the usefulness of a tibia counter rotator (TCR), a corrective orthosis based on the theory of the tibia torsional transformer, has been demonstrated, but the evidence is limited. In the present study, the efficacy and safety of TCR treatment were investigated in pediatric patients with internal tibial torsion. The subjects were 124 pediatric patients with internal tibial torsion who were between 3 and 15 years of age and had no underlying diseases. The severity of tibial intorsion was evaluated by the tibial transmalleolar angle (TMA). A TMA less than 5° was defined as internal tibial torsion, and less than −20° was defined as severe in this study. The median duration of TCR use was 11 (9, 12) (median (IQR: interquartile range)) months, and the treatment completion rate was 94.4% (117/124). The TMA at 12 months from the start of treatment in patients who completed treatment was 5° (0°, 10°) on the right (*n* = 66) (*p* < 0.01 vs. pretreatment) and 0° (−5°, 8°) on the left (*n* = 71) (*p* < 0.01 vs. pretreatment). The tibial torsional transformer used in this study is effective in the initial treatment of mild to severe internal tibial torsion, with no adverse effects. Although internal tibial torsion is generally expected to resolve spontaneously, TCR treatment may be an effective alternative to surgical therapy in the Asian pediatric population.

## 1. Introduction

Internal tibial torsion is a condition in which the axis connecting the distal tibial epiphysis and distal fibular epiphysis is medially twisted compared with the axis connecting the proximal tibial epiphysis and the distal epiphysis. This condition leads to a gait abnormality occasionally seen in children, especially causing an inner thigh gait. During the fetal period, the tibia is twisted internally due to the limited space for the fetus to move in the uterus. Subsequently, it improves spontaneously in the majority of children during postnatal development [1]. In East Asian countries with a floor-sitting culture, the number of children with internal tibial torsion is higher than in Western countries, since sitting directly on the floor disrupts the natural outward roll of the tibia [2,3,4]. Furthermore, rotating the legs inward in a prone position or sleeping with the hips and knees bent (knee-tuck position) may cause internal tibial torsion [4]. In general, children with internal tibial torsion should be followed up until approximately 8 years of age. If spontaneous healing does not occur, and severe internal torsion (internal rotation > 15°) and a severe functional or cosmetic abnormality are present, surgery should be considered [5]. However, distal tibial derotation osteotomies are fraught with complications, such as osteomyelitis, skin dehiscence, and valgus deformity. Generally, braces, night splints, shoe modification/wedges, other orthotics, and serial casting are not recommended for this condition [6].

In contrast, Bresnahan et al. [7] reported the usefulness of a tibia torsional transformer in children under 5 years of age for the initial treatment of internal tibial torsion. Later, a group in Korea developed a tibia counter rotator (TCR), a corrective orthosis based on the theory of this tibia torsional transformer. They also reported that the TCR in combination with a toe-out gait plate (GP) was effective in the treatment of internal tibial torsion [2]. However, the number of cases treated in that study was limited, and the outcomes after TCR treatment were still unclear. In the present study, the efficacy and safety of a combined treatment with the TCR and a GP in Japanese pediatric patients with internal tibial torsion were examined.

## 2. Materials and Methods

### 2.1. Methods and Patients

**Figure 1:**
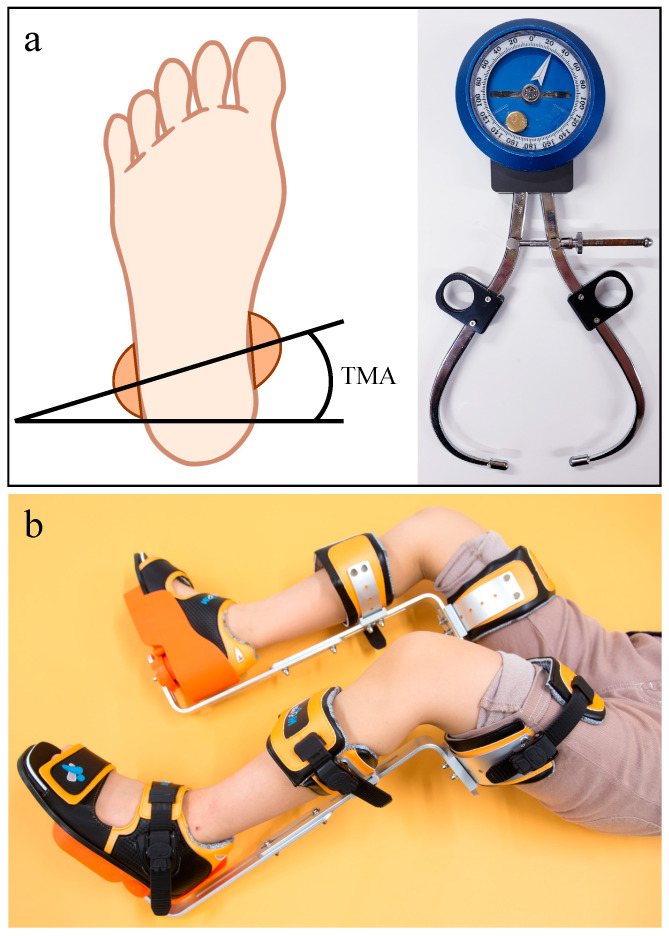
(**a**) A photo of a gravity goniometer and measurement of the transmalleolar angle (TMA). The TMA is the angle measured using a gravity goniometer [2,8] between the line connecting the points bisecting the medial tibial condyle and the line bisecting the lateral fibular condyle, while the knee is extended in the coronal plane in the supine position. Negative values indicate internal rotation of the tibia, and positive values indicate external rotation. (**b**) Photos of a tibia counter rotator (TCR). The TCR consists of padded straps that contact the thighs and lower legs, a plate with a rotating unit for foot adjustment, and metal braces. The foot plate is fixed at an angle of 15°–20° more externally rotated than the TMA of the patient.

Children who visited our clinic between December 2012 and December 2018 and were diagnosed with internal tibial torsion were included in the study. The inclusion criteria were Japanese pediatric patients who were between 3 and 15 years of age and had no underlying osteological disorders, mental retardation, or neurological diseases. The severity of tibial intorsion was evaluated by the tibial transmalleolar angle (TMA). The TMA was measured indirectly with a gravity goniometer after marking the medial malleolus of the tibia and the lateral malleolus of the fibula with the patient in the supine position with the knees extended, as previously described [2] (Figure 1a). A TMA less than 5° was defined as internal tibial torsion, and less than −20° was defined as severe in this study.

Patients were provided with a tibia counter rotator (TCR; Biomechanics Technology Co., Ltd., Goyang, Korea), which allows separate adjustments of left and right angles, to wear for at least 3 h while sleeping at night so as not to interfere with the child’s daily activities (Figure 1b). In all cases, a gait plate (GP) inserted in the shoe (Biomechanics Technology Co., Ltd.) was used in combination with the TCR. The TCR and GP were custom-made to fit each patient. The TCR used in the present study to treat tibial torsion in children consisted of an aluminum alloy skeleton weighing approximately 300–700 g, thigh and calf belts, and lower leg shoes, which were attached to the lower leg with the knee bent at 90°. In this way, the force is prevented from being transmitted to the thigh, and the corrective force is transmitted directly to the tibia. On the back of the shoe portion of the orthosis is a device with a rotation axis that can be adjusted to correct the torsion of the tibia (Figure 1b). The patient’s TMA was measured every 4 to 8 weeks, and each time, the angular device on the back of the shoe portion of the orthotic was adjusted and fixed at an angle of 15° to 20° of external rotation from the patient’s TMA. Treatment endpoints were set as the time of reaching normal values for each patient based on previous reports [8,9] and/or 12 months after the start of treatment. Patients who discontinued treatment within 3 months were defined as a putative control group (withdrawal group).

### 2.2. Statistical Analysis

Statistical analysis was performed using the Friedman test (v.8.0c; GraphPad Software, La Jolla, CA, USA) for the TMA and for changes over time following treatment initiation. *p*-Values < 0.05 were considered significant.

### 2.3. Ethics Statement

This study was approved by the Juntendo University Institutional Review Board (Approval No. 2016118, 11 January 2016) and was performed in accordance with the 1964 Declaration of Helsinki and its later amendments or comparable ethical standards (as revised in Edinburgh 2000). Informed consent was obtained from the patients and/or their parents prior to study enrollment.

## 3. Results

**Figure 2:**
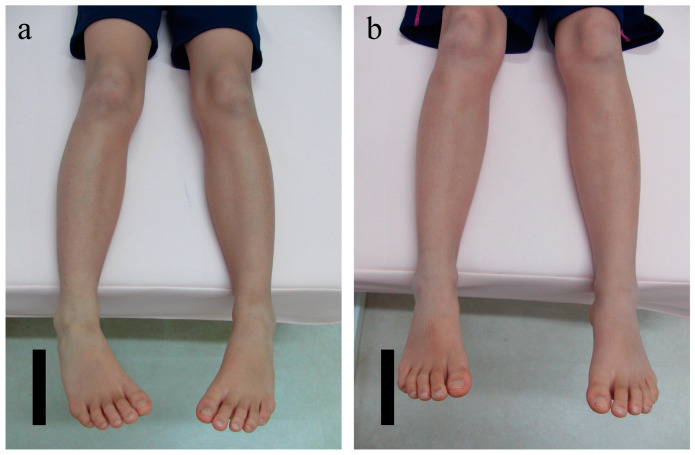
A 9-year-old boy provided with tibia counter rotator (TCR) treatment. (**a**) Pretreatment (TMA, right: −30.0°; left: −30.0°), (**b**) 12 months after pretreatment (TMA, right: 10°; left: 10°). TCR: tibia counter rotator, TMA: transmalleolar angle.

The baseline characteristics of the patients enrolled in this study are summarized in Table 1. There was no difference in the age of starting treatment between the treatment completed group and the withdrawal group (control). The median duration of TCR use was 11 (9, 12) (median (IQR: interquartile range)) months, and the treatment completion rate was 94.4% (117/124). Seven patients withdrew from treatment due to discomfort of wearing the TCR. The TMA before treatment was −20° (−20°, −15°) for the right leg and −20° (−25°, −15°) for the left leg. The TMA at 12 months from the start of treatment in patients who completed treatment was 5° (0°, 10°) on the right (*n* = 66) (*p* < 0.01 vs. pretreatment) and 0° (−5°, 8°) on the left (*n* = 71) (*p* < 0.01 vs. pretreatment) (Table 2). In the 7 patients who discontinued treatment (average wearing time: 2.5 months), no improvement in the TMA was observed. There were no adverse events associated with TCR orthotic application in any of the patients. The appearance of the lower limbs in a sample patient before and after treatment is shown in Figure 2.

## 4. Discussion

In the present study, the treatment of internal tibial torsion in children using a tibial torsion transformer was described. Internal tibial torsion is more common in the Asian population than in Western populations. Generally, surgery should be considered for the treatment of severe internal tibial torsion. A newly developed tibial torsion transformer, the TCR, is effective in the initial treatment of mild to severe internal tibial torsion. TCR treatment was carried out for Japanese children over 3 to 15 years of age with internal tibial torsion, and it was found that this condition improved after wearing the device for more than 3 h during sleep over a treatment period of approximately 11 months.

The degree of internal tibial torsion changes as the individual grows from fetus to childhood. In the fetus, the tibia is internally twisted in the uterus due to restricted mobility. After birth, the tibia undergoes external torsion, reaching a neutral state (0°) at 4–6 months of age. Thereafter, the tibia becomes externally twisted to a near adult angle until 3 years of age, and by 7 to 8 years of age, the tibia is gently twisted to the same degree as an adult (18° to 23°) [8]. Thus, this indicates that the tibia in the internal torsional position is not corrected spontaneously anymore after the age of 7–8 years. When medial tibial torsion does not improve spontaneously and becomes severe, it may cause not only cosmetic problems, but also trauma due to falls and fatigue and pain in the feet due to the combination with flat feet, thus interfering with daily life [10]. Furthermore, a close relationship between tibial torsion and osteoarthritis of the knee has been reported [3,10,11,12,13,14,15]. In a study of 1200 adult patients with osteoarthritis, Turner et al. reported the presence of uncorrected, persistent, internal tibial torsion in childhood in 65% of patients [12]. Yagi also found that the average tibial eversion in normal adults was 23.5°, and the average tibial eversion in patients with osteoarthritis of the knee was 11.3°, a significant decrease [14]. Consequently, treatment of internal tibial torsion in childhood may be effective in preventing osteoarthritis of the knee in adulthood.

In the present study, the tibial torsion angle was measured by the TMA using a gravity goniometer to assess the degree of internal tibial torsion, as previously described [8,9]. The direct methods of measuring the tibial torsion angle include computed tomography (CT) and magnetic resonance imaging [16,17], but indirect methods, such as the TMA and the tight foot angle (TFA) [18], are more appropriate for children due to radiation exposure from CT and also time and cost. Valmassy and Stanton et al. reported that the TMA in childhood and adulthood is calculated to be 5° less than the internal tibial torsion angle, with normal adults having a TMA of 13°–18° [8]. TMA measurement was an affordable and convenient method to evaluate the degree of improvement of internal tibial torsion over time in the present study.

The treatment of tibial anterolateral torsion remains controversial. In the past, the treatment approach was to wait and see until symptoms improved spontaneously. However, this approach often resulted in problems with the feet and legs in adulthood [11]. Up to now, various orthotics and plantar foot orthoses have been used to treat tibial torsion, but there has been much debate regarding their effectiveness or inappropriateness. One known treatment mechanism is to increase the degree of tibial abduction by constantly tractioning the distal tibial epiphysis with an orthotic [19,20]. That is, when stress is applied to the normal adult tibial epiphysis, the direction of bone growth changes according to Wolff’s Law, and bone remodeling occurs [21]. A typical orthosis used previously to treat tibial torsion is the Denis Browne bar [4,22]. However, the very wide Denis Browne bar has the disadvantage of potentially causing genu varum and the disadvantage of hip instability due to possible subluxation of the ankle joint.

Bresnahan and Lubert have applied the tibial torsion transformer to treat tibial torsion with good results [7]. The tibial torsion transformer is applied with the knee joint in flexion and is designed so that the corrective force is transmitted only to the distal or proximal end of the tibia. The TCR also allows independent movement of the two legs, which reportedly has the advantage of being much easier to apply than conventional braces. The TCR used in the present study has a quantified rotation angle adjustment unit attached to the sole of the shoe. This has the advantage that the angle can be easily adjusted in the clinical setting according to the patient’s tibial torsion condition, minimizing patient discomfort and adverse events. In fact, no adverse events were identified in this study. In addition, because the TCR is left and right independent, both legs are flexible, and it can be worn for extended periods of time while lying down. These advantages make it easier to maintain compliance with TCR treatment, consistent with the 94.4% (117/124) completion rate in the present study. Furthermore, since prolonged prone sleeping may be involved as a factor in tibial internal torsion [23], TCR is also expected to serve as a night splint to prevent it. The TMA of the patients who completed treatment 12 months after the start of this case study averaged 5° for the right leg (vs. pretreatment) and 0° for the left leg (vs. pretreatment) (Table 2). These results suggest that the high treatment compliance was due to the structural characteristics of the TCR, its usefulness as a night splint, and the convenience of outpatient testing by indirect measurement of tibial torsion by TMA using a gravity goniometer, allowing for minor orthotic adjustments.

A limitation of the study was that an authentic control group was not available, since the study was focused on patients whose parents requested TCR treatment. However, TMA returned to pretreatment levels in patients who had discontinued TCR placement, suggesting that TCR therapy likely has a beneficial effect on patients with internal tibial torsion. The TCR treatment used in the present study is not approved by insurance in Japan, and making the device is costly, at $1500/per person. However, future cost-effectiveness studies are needed, because this treatment may be effective from childhood to potentially prevent the development of future knee joint diseases.

## 5. Conclusions

TCR treatment was carried out for Japanese children over 3 to 15 years of age with internal tibial torsion, and it was found that this condition improved after wearing the device for more than 3 h during sleep over a treatment period of approximately 11 months. It is also meaningful that pediatricians, who routinely monitor the growth and development of children, conducted this study as the practitioners themselves and reported the effectiveness of the treatment. As the TMA is adjusted gradually with the effect of treatment, it avoids damaging the hip joint and makes it easier to maintain the patient’s compliance. In severe cases of internal tibial torsion, especially in Asian countries where there are a significant number of patients, TCR treatment is noninvasive and may be a beneficial alternative to surgery.

## Figures and Tables

**Table 1:** Patients’ characteristics.

	Completed		Control (Withdrawal)	
Number of patients	117 *		7 **	
Age, median (years)	7	(5.0, 9.0)	8	(4.3, 8.5)
Sex (male)	72		3	
Height, median (cm)	121.0	(107.7, 131.7)	125.4	(107.0, 127.4)
Weight, median (kg)	22.2	(18.4, 31.4)	26.1	(19.3, 27.9)
Treatment period, median (months)	11	(9, 12)	3	(5, 5)

Data given in parentheses are interquartile range (IQR). * Treatment was performed for both legs in 112 patients and only the left leg in 5 patients. ** All 7 patients received treatment for both legs.

**Table 2:** Change in the TMA from pretreatment to 12 months after treatment.

	Completed								Control (Withdrew)					
	Right (*n* = 112)				Left (*n* = 117)				Right (*n* = 7)			Left (*n* = 7)		
(Months)	*n*	TMA (°)	(IQR)	*p*-Value	*n*	TMA (°)	(IQR)	*p*-Value	*n*	TMA (°)	(IQR)	*n*	TMA (°)	(IQR)
Pretreatment	112	−20	(−20, −15)	-	117	−20	(−25, −15)	-	7	−20	(−28, −20)	7	−20	(−24, −18)
1	91	−10	(−15, −7)	*	95	−15	(−20, −10)	*	5	−25	(−26, −18)	5	−25	(−28, −10)
2	97	−10	(−13, −2)	*	101	−10	(−15, −5)	*	6	−18	(−23, −15)	6	−16	(−16, −11)
3–4	103	−2	(−10, 3)	*	107	−5	(−5, 0)	*	7	−15	(−20, −15)	7	−15	(−17, −9)
5–6	102	0	(−5, 5)	*	105	−5	(−10, 3)	*	5	−20	(−20, −18)	6	−12	(−19, −6)
7–8	85	5	(0, 9)	*	91	0	(−5, 5)	*	4	−20	(−20, −18)	3	−20	(−25, −20)
9–10	79	5	(0, 10)	*	83	0	(−5, 5)	*	3	−19	(−19, −14)	3	−10	(−17, −10)
11–12	66	5	(0, 10)	*	71	0	(−5, 8)	*	1	−25		2	−18	

Data for TAM (°) are presented as medians. TMA: tibia transmalleolar angle; IQR: interquartile range. * *p* < 0.01, comparison with pretreatment values.
